# In Situ Formation of ZrB_2_ and Its Influence on Wear and Mechanical Properties of ADC12 Alloy Mixed Matrix Composites

**DOI:** 10.3390/ma14092141

**Published:** 2021-04-23

**Authors:** Lalta Prasad, Niteesh Kumar, Anshul Yadav, Anil Kumar, Virendra Kumar, Jerzy Winczek

**Affiliations:** 1 Department of Mechanical Engineering, National Institute of Technology Uttarakhand, Srinagar 246174, Uttarakhand, India; laltaprasad@nituk.ac.in; 2 Department of Mechanical Engineering, GB Pant Institute of Engineering and Technology, Garhwal 246194, Uttarakhand, India; niteshk599@gmail.com; 3 Membrane Science and Separation Technology Division, CSIR—Central Salt and Marine Chemicals Research Institute, Bhavnagar 364002, Gujarat, India; anshuly@csmcri.res.in; 4 Department of Mechanical Engineering, Kamla Nehru Institute of Technology, Sultanpur 228118, Uttar Pradesh, India; anilk@knit.ac.in (A.K.); veer.iitdmech@gmail.com (V.K.); 5 Department of Technology and Automation, Częstochowa University of Technology, 42-201 Częstochowa, Poland

**Keywords:** metal matrix composites, ZrB_2_, ADC12, wear, mechanical properties

## Abstract

In this work, aluminium alloy ADC12 reinforced with various amounts of ZrB_2_ (0 wt.%, 3 wt.%, 6 wt.%, 9 wt.%) were synthesized by an in-situ reaction of molten aluminium with inorganic salts K_2_ZrF_6_ & KBF_4._ XRD, EDAX, and SEM techniques are used for the characterization of the fabricated composite. XRD analysis revealed the successful in situ formation of ZrB_2_ in the composite. From the SEM images, it was concluded that the distribution of reinforcement was homogeneous in the composites. A study of mechanical and tribological properties under the dry sliding condition of ZrB_2_-reinforced ADC12 alloy has also been carried out. It is seen that there is an increase in tensile strength by 18.8%, hardness by 64.2%, and an increase in wear resistance of the material after reinforcement. The ductility of the material decreased considerably with an increase in the amount of reinforcement. The composite’s impact strength decreased by 27.7% because of the addition of hard ZrB_2_ particulates.

## 1. Introduction

Various industries have recognized aluminium alloy metal matrix composite (MMC) as suitable material in various applications due to its property viz., strength, structural rigidity, dimensional stability, and lightweight. The MMCs provide enhanced properties over monolithic alloys. These type of materials retain their individual characteristics even if incorporated into composites and exhibit their advantages [1,2,3]. Aluminium alloys are widely used matrix materials for MMCs. The metal matrix composites are expected to be a replacement of different conventional materials in many commercial and industrial applications [2,4,5,6,7].

The aluminium alloy ADC12 is also widely known as the Japanese industrial standard alloy. ADC12 is a common die-casting material with good castability, machining characteristics, conductivity, and minimal die-casting defects [8]. The variation in properties with the addition of 2–3 wt.% in situ nano ZrB_2_-reinforced in A356 alloy was carried out by Tao et al. [9]. Their results showed that the addition influenced the distribution and size of the ZrB_2_. Dinaharan et al. [10] studied ZrB_2_-reinforced AA6061 aluminium alloy composites and found an increase in ultimate tensile strength (UTS) and hardness with an increase in ZrB_2_ content. The effect of micro and nano-size reinforcement of TiB_2_ on aluminium metal composites (AMCs) was studied by Akbari et al. [11]. They employed the stir casting method for the preparation of composites with different vol.% (0, 0.5, 1.5, 3, and 5) of TiB_2_. At elevated temperature, a comparative study of mechanical properties on AA6061/TiB_2_ and AA7015/TiB_2_ composites was performed by Oñoro [12]. The results showed that at room temperature as well as elevated temperature, the AA7015-TiB_2_ composites have higher strength than that of AA6061-TiB_2_. The modulus elasticity of the Al-Si/TiB_2_ composite in the temperature range of 25–3500 °C was studied by Han et al. [13]. They concluded that the reinforced alloy has a higher yield and tensile strength compared to unreinforced alloy at room temperature. Though, in the temperature range of 200–3500 °C, no significant increase in the yield and tensile strength was reported.

There have been few works with reinforcement of ADC12 alloys in literature. Sun et al. [14] synthesized in situ Al_3_Ti/ADC12 composite using the reaction system with the ultrasonic-assisted direct melt method. The results showed that ultrasonic chemistry reactions accelerated the formation of Al_3_Ti in comparison to the traditional in situ method. The microstructure of composites with Al_3_Ti-reinforced particles of 1–2 μm in size was found well distributed. It was also found that the tensile strength and elongation rate of sonicated composites improved in comparison to those without ultrasonication. Kakaravada et al. [15] studied A356/TiB_2_/TiC composites synthesized with a K_2_TiF_6_-KBF_4_-graphite (C) reaction system for different reinforcement ratios. They found that with the increase in volume fraction of the reinforcement, the hardness increases by 49% at 7.5% reinforcement. The 7.5% reinforced composite retained the UTS up to 84.4% and the ductility up to 27% at 3000 °C.

The practicability of the in-situ formation has been constituted in studies on metal alloys successfully reinforced in-situ ZrB_2_ particulates [16,17,18]. Kumar et al. [16] developed ZrB_2_ particles-reinforced aluminium alloy composites by an in-situ reaction of molten AA5052 alloy with two inorganic salts K_2_ZrF_6_ and KBF_4_ at a temperature of 860 °C with different volume percent (i.e., 0 vol.%, 3 vol.%, 6 vol.%, 9 vol.%, and 10 vol.%) of the reinforcement. The composites’ density and mechanical properties increase with an increase in the amount of reinforcement up to 9 vol.%. Zhang et al. [19] produced A356/ZrB_2_ AMCs using in situ reaction between K_2_ZrF_6_ and KBF_4_ and observed that the wear rate of the composites decreased with an increase in ZrB_2_ particulates reinforcement. In another study, Kumar et al. [20] investigated AA6351/ZrB_2_ in situ AMCs in which the reinforcement was produced by mixing K_2_ZrF_6_ and KBF_4_ in the molten state of base matrix alloy. The dry sliding wear behaviour was examined by varying the amount of metal reinforcement. Janudom et al. [21] investigated the feasibility study of die-casting of ADC12 aluminium alloy in a semi-solid state. The effect of reinforcement with graphene nanoplates (GNPs) and ultrasonication on microstructure and mechanical properties of ADC12 composites were examined by Xiong et al. [22]. The optimal value of GNPs reinforcement was found to be 0.9 wt.%, and optimal ultrasonic time 12 min. The hardness, the tensile strength, and the yield strength of the composite produced under the optimal condition showed improvement by 34.8%, 30.5%, and 42.7% compared with base matrix alloy.

Based on the literature reported above, it is fair to say that aluminium alloy ADC12 has a good affinity to adopt ZrB_2_ as reinforcement material. The combination of ADC12 alloy as a matrix and ZrB_2_ as reinforcement has not been studied until now. The phase analysis, mechanical, and wear behaviour of ZrB_2_-reinforced ADC12 matrix composite can provide a new dimension in material studies. In this work, halide salts K_2_ZrF_6_ (potassium hexafluoro-zirconate) and KBF_4_ (potassium tetrafluoroborate) are used for the formation of ZrB_2_ (Zirconium diboride). Aluminium alloy ADC12 is then reinforced with various amounts of ZrB_2_ (0 wt.%, 3 wt.%, 6 wt.%, 9 wt.%) by in situ reaction of molten aluminium inorganic salts. Various mechanical, physical, and wear tests, XRD, EDAX, and SEM, were used to characterize the composite.

## 2. Materials and Methods

### 2.1. Materials

The raw materials used in the present experiment are aluminium alloy ADC12 (die-cast), halide salts K_2_ZrF_6_ and KBF_4_. ADC12 was procured from M/s Balaji aluminium cast, Ludhiana). The material was received in the form of bricks. The chemical composition of the ADC12 alloy measured using the optical emission spectrometer (OES) is shown in Table 1. Salts K2ZrF6 and KBF4 were procured from M/s Sai scientific and traders, Roorkee, India.

### 2.2. Fabrication of ADC12/ZrB_2_ Composites

In the present study, the stir casting method was used for the preparation of the composite. The procedure has been explained in our previous studies in detail [23]. A pit furnace was used into which coke was placed and burnt. An A6-sized crucible was used for a pit furnace and was made up of graphite and clay. K_2_ZrF_6_ and KBF_4_ were added in molten aluminium alloy ADC12 and mixed for 30 min at 600 rpm. After 30 min, the molten metal was poured into an iron mould of the square shape, and the composite was cooled in the open atmosphere.

### 2.3. Material Characterization

XRD test was performed using X’Pert Pro, measurement range: 5° to 140°, the working conditions were 45 kV voltage and 40 mA current. SEM was performed using SEM Model ZEISS EVO 18 (Berlin, Germany). The Vickers hardness test was performed on Model—SSS-VM 50 with test loads—5–50 Kgf. A diamond indenter in the shape of a right pyramid with a square base and an angle of 136° between opposite faces was used. ASTM A370 standard [24] was used for preparing the tensile sample in which the size is 10 mm × 10 mm × 55 mm. Tensile test was performed by the HLC591 model, with a load accuracy of ±0.5% of the measured load from 2% to 100% of the capacity of the machine, displacement resolution—0.01 mm, displacement accuracy ±0.5% of the measured value of displacement, extension accuracy ±0.5% of the measured value of extension and maximum capacity 400 kN. The Charpy test was performed by machine model AIT 300 D, maximum impact energy 300 J, striking velocity 5.308 m/s, pendulum weight 21.3 kg, drop angle 140°. The sliding wear test was performed on a pin on a disc tribometer. ASTM G40 standard [25] was used for the sample preparation. Test samples were prepared with a spherical tip, and the length and diameter of samples were 32 mm and 6 mm, respectively. The samples are tested at different rpm (200, 400, and 600) and different loads (10 N, 20 N, 30 N). For all the tests, the readings were taken thrice to establish repeatability, and their average is reported in the next sections.

## 3. Results and Discussion

### 3.1. XRD Spectral Patterns of ADC12/ZrB_2_ Composites

**Figure 1:**
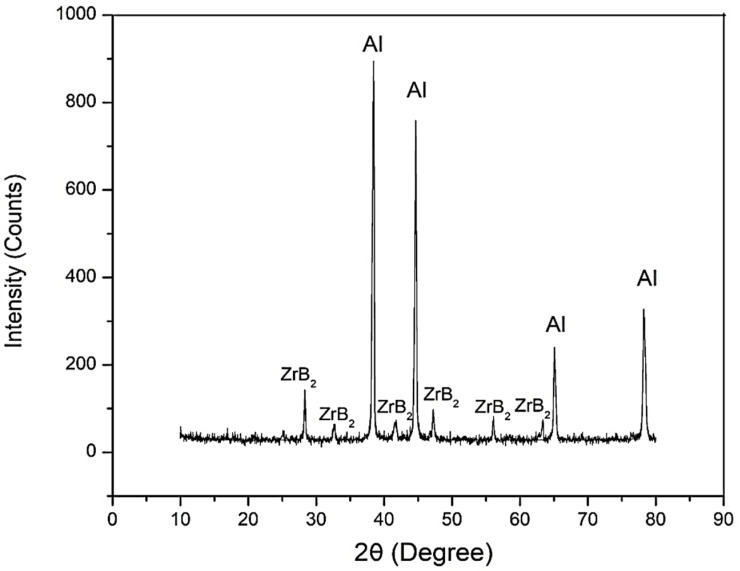
XRD of ADC12 with ZrB_2_ reinforcement.

XRD analysis is used to study the structure, composition, and physical properties of the material. The XRD spectral pattern of ADC12/ZrB_2_ composites is shown in Figure 1. The peaks which belong to ZrB_2_ particulates are visible. The chemical reactions between the molten aluminium and the inorganic salts K_2_ZrF_6_ and KBF_4_ produces ZrB_2_ particulates. From the above XRD results, it is clear that the diffraction peaks of Al_3_Zr and AlB_2_ are absent, which confirms the completion of the in-situ reaction between added inorganic salts.

### 3.2. Microstructural Study of ADC12/ZrB_2_ Composites

**Figure 2:**
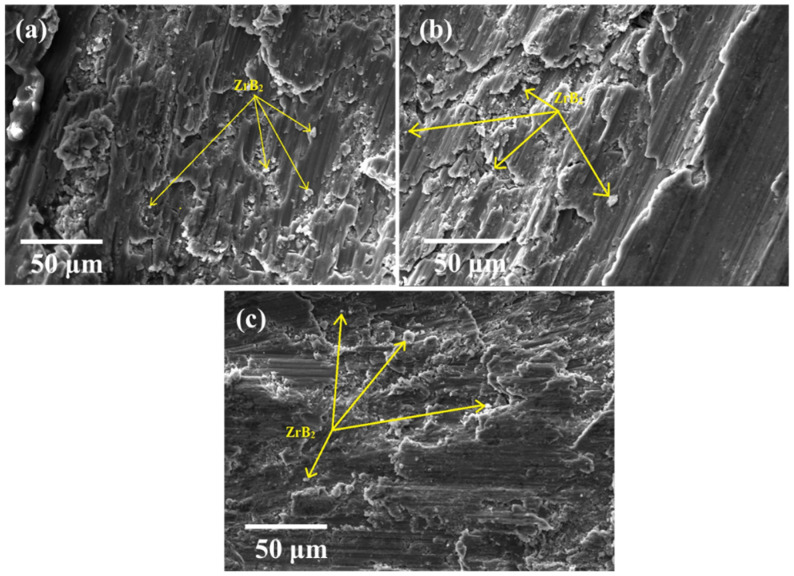
SEM micrograph of ADC12/ZrB_2_ in situ composites containing (**a**) ZrB_2_ 3%; (**b**) ZrB_2_ 6% ZrB_2_; (**c**) 9% ZrB_2_.

**Figure 3:**
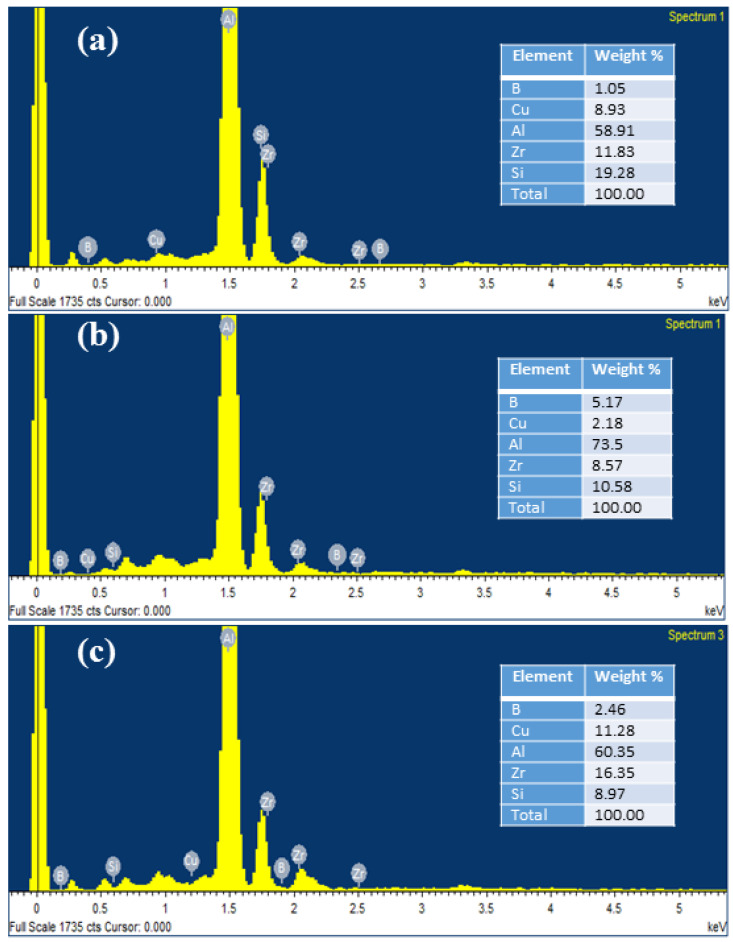
Energy spectrum produced by EDAX analysis for (**a**) 3 wt.% ZrB_2_ composites; (**b**) 6 wt.% ZrB_2_ composite; (**c**) 9 wt.% ZrB_2_ composite.

Scanning electron microscopy (SEM) is used to examine the voids in the materials, the distribution of reinforcement, and the mode of failure. The dispersion of in situ formed ZrB_2_ particulates can be seen over the aluminium matrix (Figure 2). The dispersion of reinforced particles in the alloy is almost homogeneous throughout the composites. However, the accumulation of particles at some places was also observed. The dispersion of particles within the matrix depends on many factors such as wettability of the reinforcement, stirrer speed and time of stirring, shape and size of the reinforcements. The results obtained by EDAX are presented in Figure 3. It shows the peaks of the different elements with their weight%. Since it is an aluminium alloy, the alloying constituent and Zr and B constituents can also be seen in the EDAX results.

### 3.3. Hardness Test

**Figure 4:**
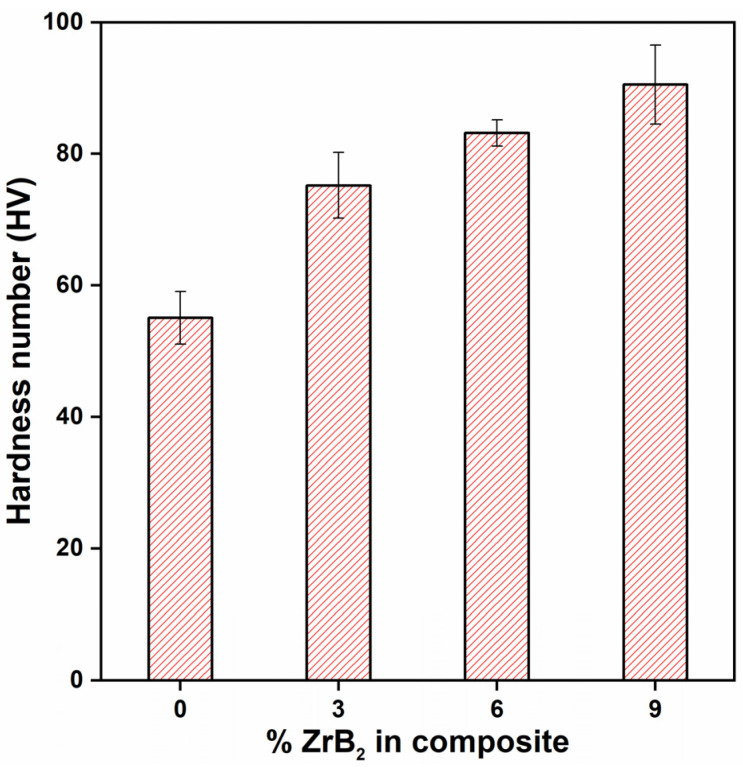
Microhardness of ADC12/ZrB_2_ in-situ composite.

Figure 4 shows the variation of microhardness in ADC12/ZrB_2_ composite for different wt.%. It is seen that the presence of ZrB_2_ particles enhanced the hardness of the composite compared with pure ADC12. During the solidification, due to different thermal expansion of ADC12 and ZrB_2,_ strain hardening occurs, increasing the hardness of the composite. Thus, grain refinement may also be a cause for increment in the hardness of the ADC12/ZrB_2_ composite [10,23]. The hardness for pure ADC12 is 55.1 HV, and with 3 wt.%, 6 wt.%, 9 wt.% ZrB_2_, the hardness is 75.1 HV, 83.1 HV, and 90.5 HV, respectively. Hence, the microhardness increases by 36%, 50.8%, and 64.2% for 3%, 6%, 9% ZrB_2_ composite, respectively, when compared to pure ADC12.

### 3.4. Sliding Wear Test

**Figure 5:**
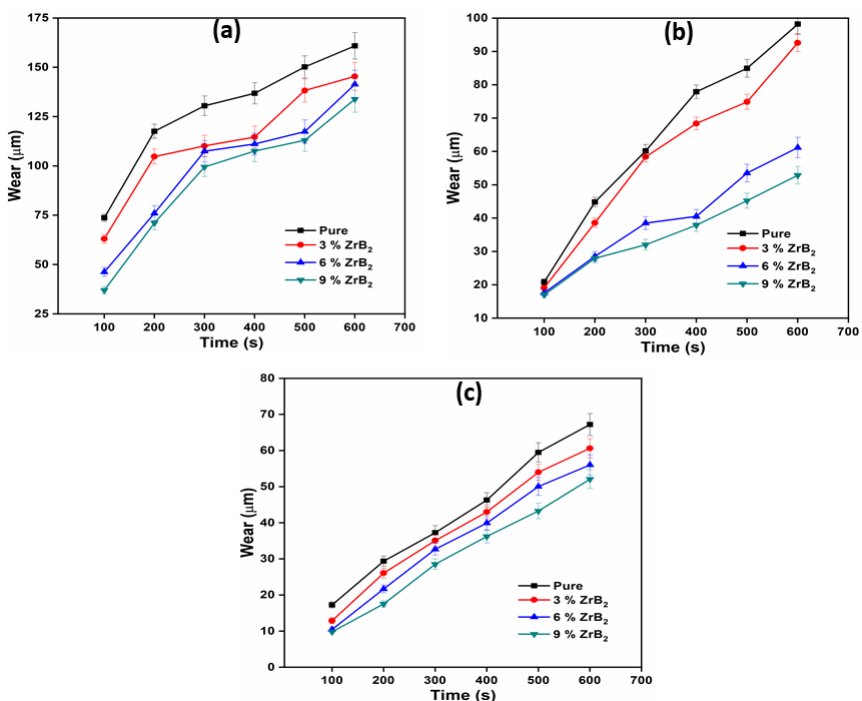
Variation of wear with time at 10N at (**a**) 600 RPM; (**b**) 400 RPM; (**c**) 200 RPM.

**Figure 6:**
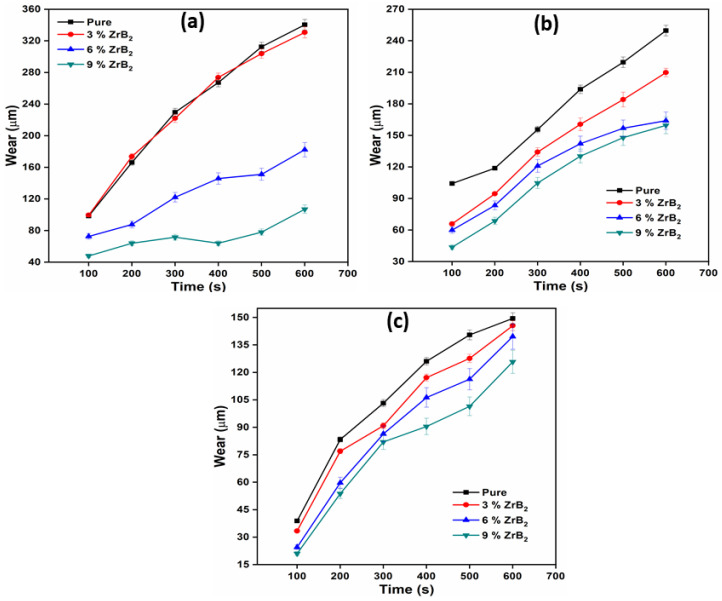
Variation of wear with time at 20N at (**a**) 600 RPM; (**b**) 400 RPM; (**c**) 200 RPM.

**Figure 7:**
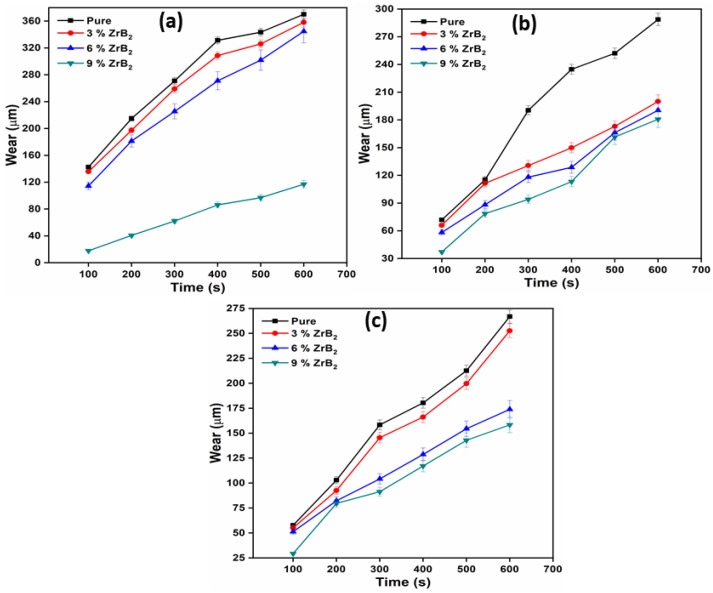
Variation of wear with time at 30 N at (**a**) 600 RPM; (**b**) 400 RPM; (**c**) 200 RPM.

A sliding wear test was performed on a pin on a disc dry sliding tribometer. The samples are tested at different rpm (200, 400, and 600) and different load (10 N, 20 N, 30 N). The wear was measured in terms of worn height (µm). The wear tests were repeated three times for each type of material composition. The average of three wear was reported at different velocity and applied normal load. Figure 5 present the worn height of pure alloy and composite at different wt.% (0, 3, 6, and 9) of reinforcement with different RPM (600, 400, 200) and a constant normal load of 10 N. Similarly, Figure 6 and Figure 7 show the worn height of pure alloy and composites at 20 N and 30 N, respectively. It has been observed that as the amount of ZrB_2_ increases, the wear resistance of the composite also increases due to high microhardness and the good interfacial bonding between the reinforcement and the matrix alloy. This is due to the presence of embedded hard ceramic particles in the soft matrix. A similar observation on wear behaviours was also reported by Kumar et al. [20,26]. The wear rate is proportional to the normal load and sliding velocity. The difference in the coefficient of thermal expansion between ADC12 and ZrB_2_ reinforcement creates a strain field at the interface due to which ZrB_2_ particles restrict the expansion of the ADC12 matrix. This will result in the enhancement of dislocation density. This increment in dislocation density increases the hardness and wear resistance of the composites. As the load and speed are increases, friction between pin and disc also increases. This is the only reason behind the higher rate of material wear [23]. The nature of wear at low load and low sliding velocity was the combination of abrasion and delamination, whereas adhesion and softening/melt wear was observed at higher load and high sliding velocity.

### 3.5. Tensile Test

**Figure 8:**
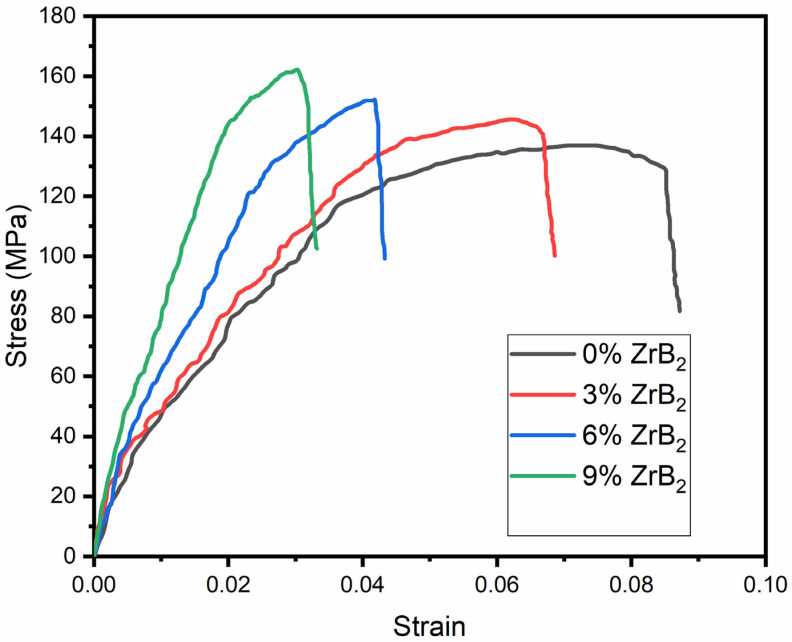
Tensile strength of ADC12 and its composites with ZrB_2_ reinforcement.

The tensile test was performed for determining the ultimate tensile strength (UTS) and yield strength (YS) of the material. It can be seen that the tensile strength of the material increased with an increase in the reinforcement of ZrB_2_ particles, whereas the ductility of the composite decreased, as seen from the stress-strain plot presented in Figure 8. Several strengthening mechanisms can be used to discuss the behaviours of the composites under tensile loading. Dislocation loads around the ZrB_2_ particles occur in the composites, which create the dislocation forest. Interaction between two dislocations is repulsive; hence, the materials’ dislocation will produce back stress for the further dislocation movement. Therefore, immense stress is required to cross through the dislocation forest. Ultimately it increases the strength of the material. According to the Hall–Petch relationship, grain refinement by ZrB_2_ can also provide additional strength to the material [10]. It is a method in which strength is provided by reducing average particle size. Whenever there is grain refinement, by applying the load, dislocation reaches the grain boundary; since atoms on the other side are oriented slightly, larger stress is required by dislocation to change its coarseness. This increases the strength of the material [27]. The ultimate tensile strength of the material increases by 6.8%, 11.4%, and 18.8% for 3%, 6%, and 9% ZrB_2_ composite, respectively, as compared to pure ADC12, as shown in Table 2.

### 3.6. Impact Test

**Figure 9:**
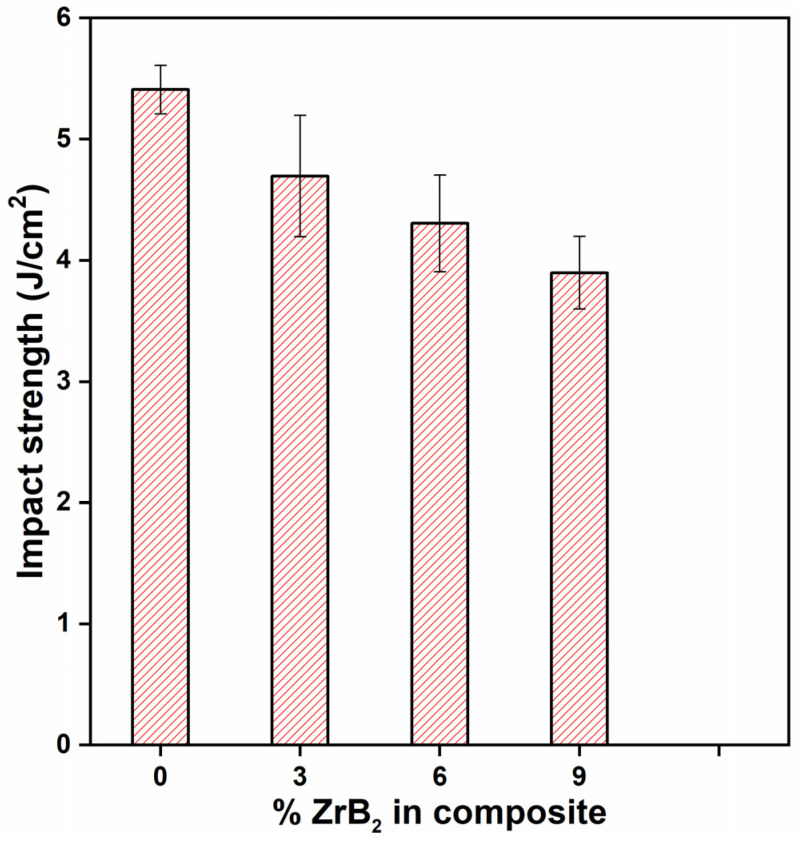
Impact strength of material with reinforcement.

The Charpy test was performed for determining the impact strength of the material. The results obtained from the test are shown in Figure 9. The impact strength values indicate that impact strength decreases with an increase in ZrB_2_ concentration compared to the base alloy ADC12. Distributing hard particles in soft material reduces the material absorption capability of the material [28]. The more the dispersion of ZrB_2_ particles, the lesser will be the impact strength. Whenever there is an impact, the bonding between the particles and the matrix breaks, due to which new surfaces will appear, and energy will release. Impact strength of ADC12 is 5.4 J/cm^2^ and but with addition of 3, 6, 9 wt.% of ZrB_2,_ impact strength reaches to 4.7 J/cm^2^, 4.3 J/cm^2^, 3.9 J/cm^2^, respectively. The impact strength of the composite material as compared to pure ADC12 is decreased by 12.9%, 20.3%, and 27.7% on addition of 3%, 6%, and 9% wt. of ZrB_2_ reinforcement, respectively. The impact strength of the composites decreases as the wt.% of the reinforcement increases due to the presence of hard ceramic particles. Similar observation also reported by previous researchers [29,30,31]. The de-bonding of ceramic reinforcement and matrix occurs easily during impact testing. As the amount of reinforcement increased, the porosity also increased, the presence of porosity may also be the reason behind the decrease in impact strength of the composites.

## 4. Conclusions

In this work, the mechanical and tribological properties of ZrB_2_-reinforced ADC12 with different wt.% were evaluated. The significant conclusions based on the above study are

- ADC12/ZrB_2_ MMC having different content (0, 3, 6, and 9 wt.%) of ZrB_2_ particulate were fabricated via in situ reaction between melted aluminium and the salts K_2_ZrF_6_ and KBF_4_. The XRD results confirmed the successful formation of ZrB_2_ particulates;
- The microstructure of the composite showed homogeneous dispersion of ZrB_2_ particles. The spectrum produced by the EDAX shows the composite elemental composition in which the Zr and B constituents are present;
- Microhardness of the MMC increases by 36%, 50.8%, and 64.2% for 3%, 6%, 9% ZrB_2_ reinforcement, respectively, as compared to pure ADC12;
- The wear test concluded that with an increase in the reinforcement, the wear resistance of the material increased, and as the load increases wear rate also increases;
- The tensile test shows that the material’s tensile strength increased with the increasing amount of reinforcement with the reduction in ductility. The ultimate tensile strength of the material increases by 6.8%, 11.4%, and 18.8% for 3%, 6%, and 9% ZrB_2_ composites, respectively, as compared to pure ADC12;
- As the amount of reinforcement increases, the material’s impact strength decreased because of the mixing of hard particles in the soft matrix. Impact strength of the material decreased by 12.9%, 20.3%, and 27.7% for 3%, 6%, 9% ZrB_2_ reinforcement, respectively, as compared to pure ADC12.

Thus, it may be stated that the aluminium alloy composites have prospective applications in automobile industries, such as brakes and engine cylinders. From this research, it can be concluded that the in-situ reinforcement of ZrB_2_ in ADC12 can enhance its mechanical wear behaviour and also appropriate for applications in the transportation and defence industries.

## Figures and Tables

**Table 1:** Chemical composition of ADC12 alloy.

Si	Zn	Cu	Fe	Mn	Mg	Ni	Al
10.5	2.2	1.5	1.3	0.11	0.09	0.88	Bal

**Table 2:** Obtained strength for the composite.

Composition	Yield Strength (MPa)	Ultimate Tensile Strength (MPa)
ADC12	90.54	136.34
3% ZrB_2_	110.52	145.66
6% ZrB_2_	121.36	152
9% ZrB_2_	138	161.98

## Data Availability

The authors confirm that the data supporting the findings of this study are available within the article.
